# Left Atrial Appendage Closure: A Safe and Effective Alternative to Anticoagulation?

**DOI:** 10.19102/icrm.2019.100107

**Published:** 2019-01-15

**Authors:** Margaret M. Infeld, Daniel N. Silverman, Daniel L. Lustgarten

**Affiliations:** ^1^University of Vermont Medical Center, Burlington, VT, USA; ^2^University of Vermont College of Medicine, Burlington, VT, USA

**Keywords:** Anticoagulation, atrial fibrillation, left atrial appendage closure, left atrial appendage occlusion, WATCHMAN

## Abstract

To date, left atrial appendage closure (LAAC) devices continue to be assessed as an intuitive alternative to oral anticoagulant therapy to prevent embolic complications in patients with atrial fibrillation. Concerns remain about the up-front risks associated with device implantation as well as device efficacy in preventing embolic events as compared with anticoagulation. Currently, LAAC devices serve as a potential alternative to long-term anticoagulation with the benefit of decreased bleeding risk but with less protection against ischemic events. An individualized risk–benefit analysis with regard to stroke possibility, bleeding likelihood with long-term anticoagulation, the risks of an invasive procedure, and the risks associated with having a lifelong intracardiac device should be performed to guide careful patient selection for this operation.

## Introduction

The association between atrial fibrillation (AF) and stroke is well-established, as is the efficacy for reducing stroke with oral anticoagulant (OAC) therapy.^1,2^ On the basis of largely post-hoc analyses, the left atrial appendage (LAA) is believed to be the major source of thrombus leading to AF-associated embolic strokes.^3,4^ Currently, LAA closure (LAAC) devices are being assessed in an ongoing manner as an intuitive alternative to OAC therapy to prevent embolic events while lessening the risk of hemorrhagic complications. However, there are concerns about the upfront risks associated with device implantation as well as device efficacy in preventing embolic events as compared with anticoagulation.

Percutaneous approaches to LAAC include implantable devices that occlude the LAA orifice, such as the WATCHMAN™ device (Boston Scientific Corp., Natick, MA, USA) and the AMPLATZER™ Amulet™ (Abbott Laboratories, Chicago, IL, USA) as well as the LARIAT^®^ device (SentreHEART, Redwood City, CA, USA), a soft-tissue snare that cinches the LAA epicardially. In the United States, the WATCHMAN™ device (Boston Scientific Corp., Natick, MA, USA) is currently the only commercially available LAAC device and gained Food and Drug Administration (FDA) approval following results from two multicenter randomized control trials (RCTs)^5,6^ and their associated continued access registries.^7,8^ The FDA indication for this device is to reduce the risk of thromboembolism from the LAA in patients with nonvalvular AF (NVAF) who (1) are at increased risk for stroke and are recommended for anticoagulation therapy, (2) do not have a contraindication to warfarin, and (3) have an appropriate reason to seek a nonpharmacologic alternative to warfarin.^9^

## The road to Food and Drug Administration approval

The first Watchman RCT, the Watchman Left Atrial Appendage System for Embolic Protection in Patients With Atrial Fibrillation (PROTECT AF) study, randomized 707 patients with NVAF and a CHADS_2_ score of 1 or greater in a 2:1 ratio to either a WATCHMAN™ (n = 463) group or a warfarin (n = 244) group. The use of the WATCHMAN™ device (Boston Scientific Corp., Natick, MA, USA) was found to be noninferior to warfarin for the primary composite efficacy endpoint of stroke, systemic embolization (SE), and cardiovascular/unexplained death, both at 1,065 (mean: 1.5 years) and 2,621 patient-years (mean: 3.8 years) of follow-up.^5^ This finding was driven by lower rates of hemorrhagic stroke and fatal hemorrhagic stroke in the device arm. The hemorrhagic stroke incidence was 10 times higher in the warfarin arm than in the device arm at 1.5 years (2.5% versus 0.2%) and 3.8 years (4% versus 0.6%).^10^ Additionally, the rate of cardiovascular/unexplained death was lower in the device arm, with an absolute risk reduction (ARR) of 5.3% at 3.8 years. This was driven by the lower rate of fatal hemorrhagic stroke in the warfarin arm (p = 0.004), as eight of the 10 hemorrhagic strokes were fatal.^5^

Notably, several issues were raised with regard to the hemorrhagic stroke signal in PROTECT AF. FDA reviewers noted an uneven adjudication of hemorrhagic stroke between the study arms.^11^ Of the 10 hemorrhagic stroke events in the warfarin arm, five occurred after falls, four were associated with a subdural hematoma (SDH), and one was associated with a subarachnoid hemorrhage. In current guidelines, hemorrhagic stroke is by definition not caused by trauma.^12^ In the WATCHMAN™ arm, three subjects also fell and had SDH; however, these cases were categorized as intracranial bleeding events rather than hemorrhagic stroke. In total, there were five reported intracranial bleeding events in the WATCHMAN™ group and one in the warfarin group. Combining the reported hemorrhagic strokes with intracranial bleeds, there were seven events in the WATCHMAN™ group and 11 events in the warfarin group.^11^

Although in the study protocol, hemorrhagic stroke diagnosis required imaging confirmation, one control subject did not have imaging confirmation, but the reporting physician deemed it likely she had experienced such.^11^ Another control arm patient, although appropriately included in the intention-to-treat analysis, had been off warfarin for more than 38 months at the time of the hemorrhagic stroke and was taking acetylsalicylic acid (ASA) alone.^11^ Four control group patients were taking ASA in addition to warfarin at the time of the hemorrhagic event, making it difficult to assess the contribution of the concomitant antiplatelet (APT) medication. Lastly, the hemorrhagic stroke incidence in the warfarin arm of PROTECT AF, at 1.1 per 100 patient-years, was at least two times higher than the hemorrhagic stroke risk of warfarin in contemporary OAC trials, where the incidence has been consistently 0.4 to 0.5 per 100 patient-years.^13–16^

To this end, the signal toward reduced hemorrhagic stroke with the WATCHMAN™ device (Boston Scientific Corp., Natick, MA, USA) is attenuated by the inconsistent adjudication of hemorrhagic stroke events, the lack of imaging confirmation in one subject, the concomitant use of APT medication in several subjects with hemorrhage, the small sample size leading to potentially spurious results, and the significantly higher rate of hemorrhagic stroke in the warfarin arm as compared with in recent OAC trials.^11^ The lower rate of cardiovascular/unexplained death, fatal hemorrhagic stroke, and overall lower hemorrhagic stroke rate in the WATCHMAN™ arm was not reproduced in the later Evaluation of the WATCHMAN™ LAAC Device in Patients with AF versus Long-term Warfarin Therapy (PREVAIL) trial. The hemorrhagic stroke rate in the PREVAIL control group (0.67 per 100 patient-years) was in the range observed in the warfarin groups of contemporary OAC trials.^6^

Embolic event rates are the usual comparator in AF therapies and have been consistently higher with use of the WATCHMAN™ device (Boston Scientific Corp., Natick, MA, USA) versus with warfarin. The rates of ischemic stroke for the device and drug arms were 5.2% (24/463) versus 4.1% (10/244) at 3.8 years, and three SE events occurred in the device arm only. Combining ischemic stroke and SE events, warfarin had an ARR of 1.7% at 3.8 years. Early in the PROTECT AF study, several ischemic strokes were caused by air emboli, which improved with operator experience.^5–8^ When excluding procedural ischemic events, the ischemic events in PROTECT AF were not statistically different between the WATCHMAN™ and warfarin groups.^10^ However, skepticism was raised regarding the low-risk patient population. The average CHADS_2_ score was just 2.2 and more than 30% of patients had a CHADS_2_ score of 1, which may have made it easier to establish noninferiority for the composite endpoint.^5^ There was also concern that the concomitant use of antithrombotic medications and indefinite ASA played a role in the already modest efficacy observed in the WATCHMAN™ arm.

Although the FDA had reservations about efficacy, the main concern was the early adverse event rate of 8.7% within seven days of implant. Because of safety events that occurred early on in the trial, use of the WATCHMAN™ device (Boston Scientific Corp., Natick, MA, USA) was deemed inferior to warfarin in terms of the primary safety endpoint, a composite of major bleeding or procedure-related complications, at 1.8 years, but it did meet noninferiority at 3.8 years.^17^ The PREVAIL trial was the second RCT to consider the WATCHMAN™ device (Boston Scientific Corp., Natick, MA, USA) and was designed in part to address these concerns. The focus of PREVAIL was to demonstrate that safety improvements observed during the second half of the PROTECT AF trial could be reproduced and successfully adopted by new centers. PREVAIL had similar enrollment criteria as those of the PROTECT AF study but included higher-risk patients, presented a mean CHADS_2_ score of 2.6, and excluded patients with an indication for long-term clopidogrel therapy.^6^ Bayesian statistical methods allowed an efficacy composite to be studied in what was planned to be a smaller trial incorporating PROTECT AF data with a discounted weight of 50%.

As it turned out, warfarin was superior to the WATCHMAN™ device (Boston Scientific Corp., Natick, MA, USA) for the PREVAIL trial’s composite primary efficacy endpoint of stroke, SE, and cardiovascular/unexplained death. Patients using the device had higher rates of each of the following events in comparison with those using warfarin: ischemic stroke (1.9% versus 0.7%), hemorrhagic stroke (0.4% versus 0%), death (2.6% versus 2.2%), and SE (0.4% versus 0%). The total number of events, however, was still very small; for example, the number of ischemic strokes/SE in the device arm was six of 269, versus one of 138 in the warfarin arm. The second primary efficacy endpoint in PREVAIL was “late” ischemic stroke or SE occurring at greater than seven days after randomization. This was intended to answer the proof-of-concept questions, “is the LAA *the* source of embolism in NVAF?” and “do LAAC devices offer the same protection from ischemic events as OAC *after* overcoming the procedural risks?”^10^ Although the late ischemic event signal was higher in the device group (2.53%) versus the drug group (2%), the 18-month risk difference was statistically noninferior.^6^

In the July 2014 publication of PREVAIL, only 28% of patients were reported to have reached the planned 18-month follow-up point. Updated data released later revealed eight additional ischemic strokes in the device arm, for a total of 14 ischemic events (5.2%), versus one event (0.7%) in the warfarin arm. Of the 14 ischemic events, only one was caused during the procedure^18,19^; thus, these new results suggested that the WATCHMAN™ device (Boston Scientific Corp., Natick, MA, USA) was now inferior to warfarin for the proof-of-concept late ischemic endpoint.^11^ The trialists suggested that PREVAIL was not powered to detect differences in ischemic stroke and pointed to PREVAIL’s overperforming warfarin arm (0.34 events/100 patient-years) as the reason for the difference.^10^ However, when comparing the ischemic stroke risk seen with the WATCHMAN™ device (Boston Scientific Corp., Natick, MA, USA) to that with the warfarin arms of contemporary OAC trials with similar CHADS_2_ scores, the risk with device use was still greater at 2.3 events per 100 patient-years versus between one event and 1.4 events per 100 patient-years for warfarin.^11,13,15,16^

A patient-level meta-analysis combining the two RCTs further assessed the signals of increased ischemic stroke in the WATCHMAN™ (n = 732) arm as compared with in the warfarin arm (n = 382).^8^ The meta-analysis results were unsurprisingly similar to the results of the 3.8-year follow-up of the PROTECT AF trial, which provided the majority of patient data. At a mean follow-up of four years, the pooled primary composite endpoint met noninferiority. Participants in the device arm had higher rates of ischemic stroke and SE (45/732; 6.1% versus 14/382; 3.6%), but the difference was not statistically significant. The difference in hemorrhagic stroke was, however, significant in a manner that favored the device arm (5/732; 0.6% versus 13/382; 3.4%; p = 0.002). Notably, the hemorrhagic stroke events were driven entirely by PROTECT AF; thus, the concerns about the hemorrhagic stroke signal raised by the FDA still apply here.^11^ In addition, there were significant differences in disabling stroke (1.8% versus 3.9%; p = 0.03) and all-cause mortality (14.5% versus 19.1%; p = 0.02), driven by the difference in fatal hemorrhagic stroke in PROTECT AF, as well as in nonprocedural major bleeds (6.5% versus 13.3%; p = 0.003), respectively, favoring the device. When including procedural bleeding, major bleeds were similar between the groups.^17,19,20^

Despite the signal toward increased ischemic stroke/SE with use of the WATCHMAN™ device (Boston Scientific Corp., Natick, MA, USA), PREVAIL was not powered to demonstrate individual efficacy endpoints and the meta-analysis did not show a statistical difference. The prime directive for PREVAIL was to satisfy the safety concerns raised in PROTECT AF, which it successfully did; specifically, the composite of early procedure-related complications decreased to 4.2% in PREVAIL.^6^ This improvement and the totality of data from the RCTs was sufficiently convincing enough that the third FDA panel voted in favor of approval for the WATCHMAN™ device (Boston Scientific Corp., Natick, MA, USA) in March 2015 and the Centers for Medicare and Medicaid Services subsequently agreed to pay for the procedure.^21^ The most frequent serious complication seen in PROTECT AF, cardiac tamponade, improved from a rate of 4.3% in PROTECT AF to 1.9% in PREVAIL and then to 1.02% in safety monitoring data following FDA approval of the device.^5,6,10,22^ The rates of procedure-related stroke (0.18%), device embolization (0.25%), and procedure-related mortality (0.06%) also improved significantly.^10,17,19,20^ It was clear that increased experience and training preparation improved procedural risks.

## Stroke severity difference in left atrial appendage closure versus warfarin

While the incidences of all-cause, ischemic, and hemorrhagic stroke were similar between patients using the WATCHMAN™ device (Boston Scientific Corp., Natick, MA, USA) and warfarin, those using the former had less fatal or disabling strokes as compared with those using warfarin, with disabling strokes being defined by a change in the modified Rankin Scale score of greater than or equal to 2.^19^ This was driven by the higher number of hemorrhagic strokes adjudicated to the warfarin arm, as hemorrhagic strokes have been, in general, associated with greater disability and mortality as compared with ischemic strokes.^23,24^ Aside from the hemorrhagic stroke adjudication issues and the possibility of a spurious result based on a small sample size, patients with a reason to try an alternative to anticoagulation (eg, higher bleeding risk) may have been preferentially referred by their physicians or self-referred in response to study announcements. This may be generalizable to the real-world population of patients considering alternatives to long-term anticoagulation.

## Difficulties encountered with left atrial appendage closure devices at our institution

In addition to the continued evaluation of LAAC efficacy, upfront and long-term risks of LAAC placement should be considered in the risk–benefit analysis. We present three cases from our experience, two involving the WATCHMAN™ device (Boston Scientific Corp., Natick, MA, USA) and one involving the AMPLATZER™ Amulet™ (Abbott Laboratories, Chicago, IL, USA), respectively, which highlight both early and late complications.

### Case 1

A 79-year-old male with chronic lymphocytic leukemia, chronic thrombocytopenia, bioprosthetic aortic valve replacement for aortic stenosis, and persistent AF with a CHA_2_DS_2_-VASc score of 3 was deemed an appropriate candidate for a WATCHMAN™ device (Boston Scientific Corp., Natick, MA, USA) due to his persistent thrombocytopenia and indication for long-term OAC. He was anticoagulated with apixaban, underwent screening via transesophageal echocardiography (TEE), and had a 30-mm version of the device implanted. His postoperative TEE scan showed a well-seated device without peridevice leak.

**Figure 1:**
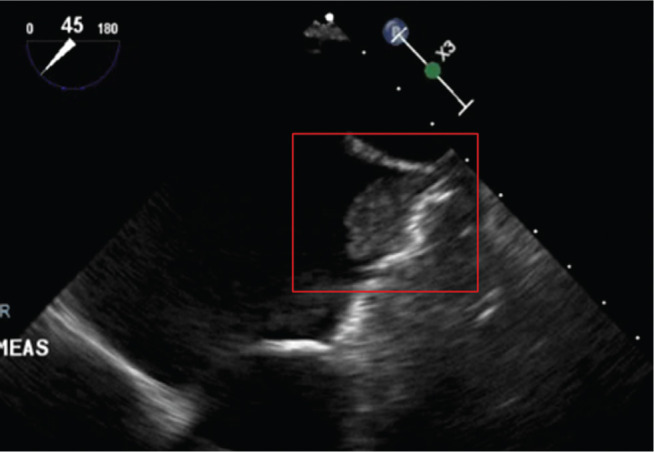
Identification of a 1.8-cm × 1.38-cm fixed vegetation on the WATCHMAN™ device (Boston Scientific Corp., Natick, MA, USA) (enclosed in red box).

**Figure 2:**
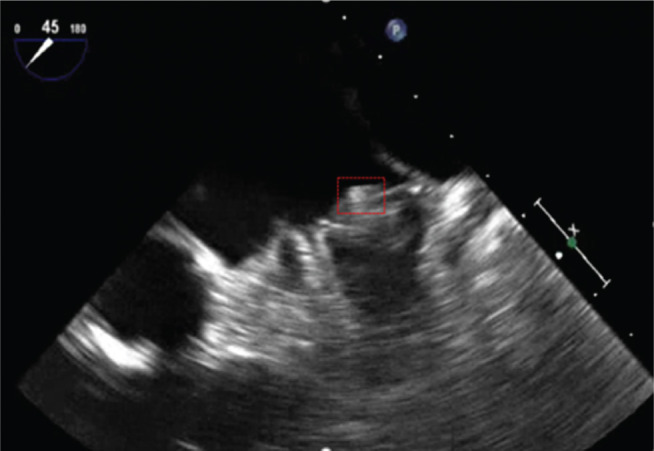
Follow-up imaging of the WATCHMAN™ device (Boston Scientific Corp., Natick, MA, USA) after one month of therapeutic anticoagulation (enclosed in red box).

One year later, the patient presented with fevers and malaise and was found to have *Streptococcus mitis* bacteremia. A TEE scan revealed a 1.8-cm × 1.4-cm fixed vegetation on the WATCHMAN™ device (Boston Scientific Corp., Natick, MA, USA) **(Figure 1)**, without evidence of valvular involvement. The vegetation appeared to be a chronic, well-organized thrombus, but clot with superimposed infection could not be excluded. The patient was treated with six weeks of antibiotic therapy; his anticoagulation was resumed; and he underwent a repeat TEE scan after six weeks, which revealed a persistent 9-mm × 6-mm vegetation **(Figure 2)**.

The patient remained in a state without subjective fevers, chills, or malaise. He was continued on anticoagulation, and repeat blood cultures were negative. Repeat echocardiogram showed that the echodensities on the device had significantly decreased in size but that new, severely increased gradients across his prosthetic aortic valve were present, without evidence of vegetation. At the time of this writing, he was undergoing evaluation for aortic valve-in-valve replacement.

### Case 2

**Figure 3:**
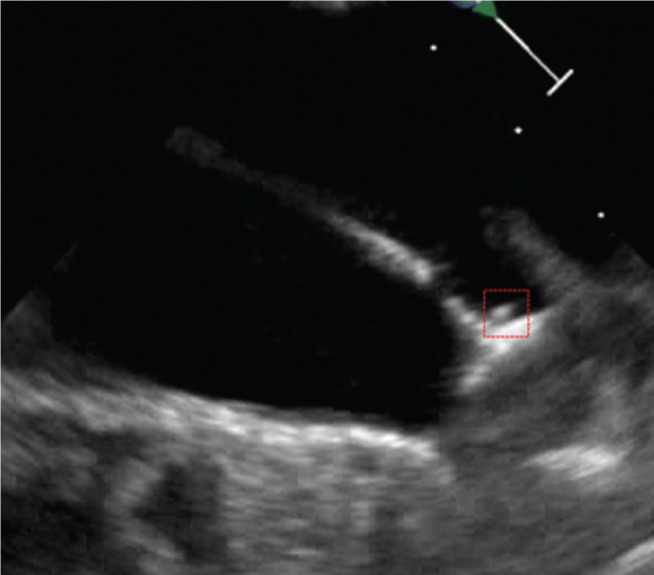
During deployment, with the thrombus partially visible on the atrial side of the device (enclosed in red box).

**Figure 4:**
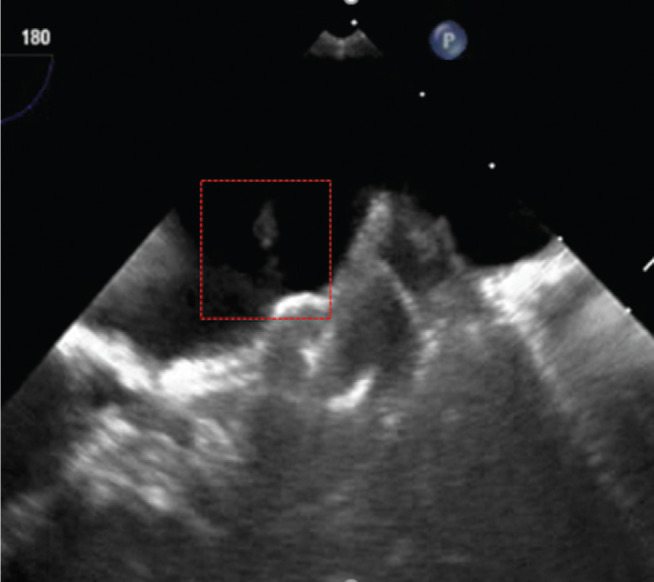
Following deployment, with the thrombus visible (enclosed in red box).

An 82-year-old female with paroxysmal AF and a CHA_2_DS_2_-VASc score of 4 was identified as an appropriate candidate for a LAAC device because of recurrent bleeding on systemic anticoagulation. She was restarted on short-term apixaban and underwent implantation of an AMPLATZER™ Amulet™ device (Abbott Laboratories, Chicago, IL, USA). During the procedure, a poorly formed thrombus was noted on the delivery device under ultrasound **(Figure 3)**. The activated clotting time (ACT) was 311 seconds, with an international normalized ratio of 1.0. A postprocedure TEE scan revealed a well-seated device with excellent seal and no peridevice flow according to color Doppler. However, a small, mobile thrombus was seen on the atrial surface of the device (ie, the disc) **(Figure 4)**.

She was admitted to the hospital and started on a therapeutic heparin infusion. A repeat TEE scan the following day showed resolution of the device-related thrombus and no peridevice leak. She was discharged on clopidogrel 75 mg and ASA 81 mg daily. Her 45-day postimplantation TEE scan showed no thrombus, but did demonstrate a small gap with a 3-mm to 4-mm diameter flow at the anterolateral aspect of the closure device with a possibility of subtle device migration. She remained on ASA and clopidogrel and has done well clinically thus far with regular cardiology follow-up.

### Case 3

**Figure 5:**
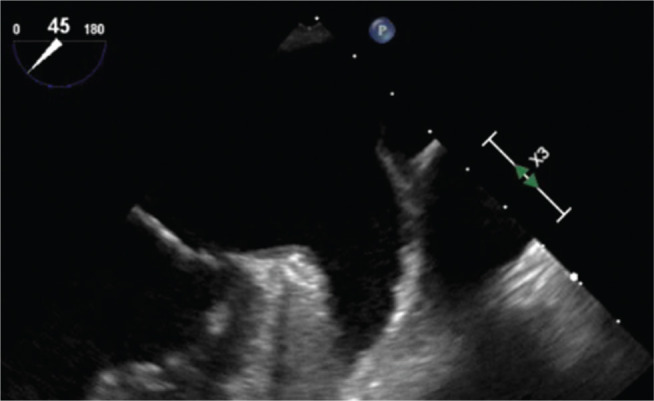
Pre-implantation, with dilated left atrium but no evidence of LAA thrombus.

**Figure 6:**
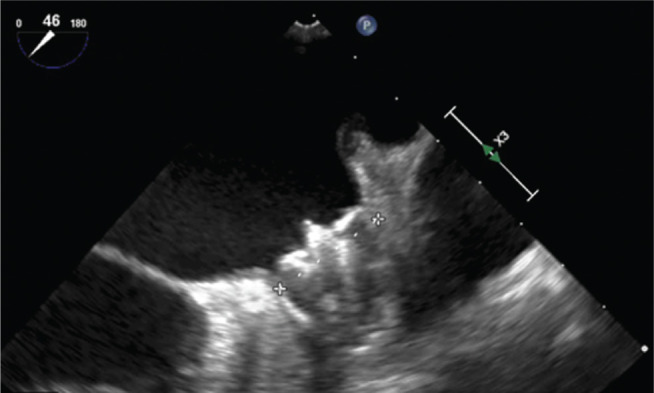
Immediately postimplantation, with a well-seated device.

A 78-year-old female with paroxysmal AF and a CHA_2_DS_2_-VASc score of 8 was referred for a LAAC device due to severe frailty and recurrent falls despite physical therapy. Her preimplantation TEE scan showed a dilated left atrium and no evidence of LAA thrombus **(Figure 5)**. She underwent implantation of a WATCHMAN™ device (Boston Scientific Corp., Natick, MA, USA) without complication, and her postprocedural TEE scan showed a well-seated device without leak **(Figure 6)**.

**Figure 7:**
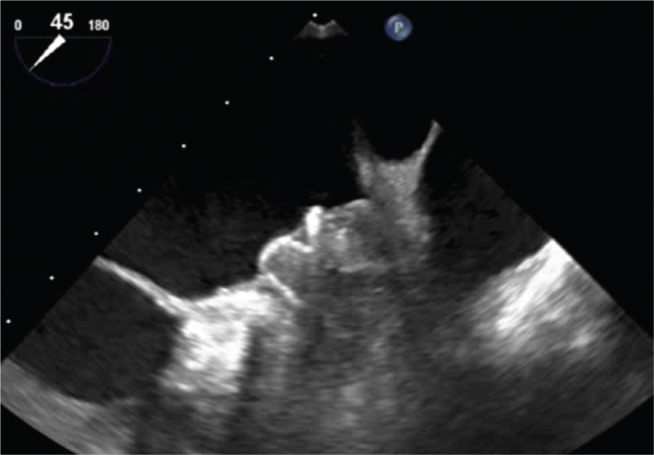
Device at follow-up with only a narrow 1-mm gap and no thrombus visible.

One month later, she was admitted for acute right monocular vision loss lasting several hours. Computed tomography brain scan findings were unremarkable. She was evaluated by ophthalmology and neurology, who suspected a central retinal artery occlusion secondary to an embolic event. TEE scan revealed a well-positioned WATCHMAN™ device (Boston Scientific Corp., Natick, MA, USA) with a maximum gap of 1 mm **(Figure 7)** and a small atrial septal defect attributed to procedural sequelae. No thrombus was identified, but imaging demonstrated a moderate burden of atherosclerotic plaque in the ascending aorta and carotid arteries. The overall impression of her clinical course was a likely embolic event of vascular or cardiac etiology, the latter possibly occurring prior to complete endothelialization of her WATCHMAN™ device (Boston Scientific Corp., Natick, MA, USA).

## High standards for left atrial appendage closure

Given the current evidence for LAAC efficacy in preventing embolic events, which is equivocal at best; the up-front procedural risks; and the long-term risk of a foreign object in the left atrium, there should be high standards in place regarding when to offer LAAC devices as an alternative to anticoagulation. Although the procedural complication rate has significantly improved, the upfront procedural risk is still 2.2% to 4.6%.^6^ The hope is that the frequency and intensity of early adverse events will continue to improve with more operator experience and successful training protocols.^25^ As seen with cases from our institution, late infectious, thrombotic, and embolic complications are also possible and justify careful adherence to device labeling as well as routine postprocedure LAA imaging.

Patients with CHA_2_DS_2_-VASc scores of 2 or greater derive an overall benefit from the stroke risk reduction associated with systemic anticoagulation^26^; thus, OAC is recommended for most patients at this risk level. The risk for ischemic stroke/SE with the WATCHMAN™ device (Boston Scientific Corp., Natick, MA, USA) implantation was 1.5% per year for patients with a mean CHA_2_DS_2_-VASc score of 3.6.^8^ Although warfarin was superior to this, with a 0.9% annual risk,^8^ the predicted stroke risk for patients without any therapy was 3.6% annually for this risk group. Obfuscating the idea that the WATCHMAN™ device (Boston Scientific Corp., Natick, MA, USA) is better than no therapy for patients with contraindications to OAC or APT medications, the device has only been studied in RCTs as part of a treatment package with warfarin and APT medication and indefinite ASA. Similarly, the manufacturer of the AMPLATZER™ Amulet™ device (Abbott Laboratories, Chicago, IL, USA) recommend dual antiplatelet therapy followed by ASA indefinitely. Part of the stroke benefit of the WATCHMAN™ device (Boston Scientific Corp., Natick, MA, USA) in comparison with imputed placebo analysis may be due to postimplantation antithrombotic therapy. Patients unable to take OAC or APT medications peri-implant will likely have an increased risk of device-related thrombus. Additionally, patients with decreased left ventricular function, stroke history, and/or contraindications to OAC are more likely to have thrombi outside of the LAA,^27^ for which LAAC provides no benefit.

A retrospective cohort study in France highlighted the issues with LAAC devices in real-world, high-risk patients not maintained on the antithrombotic regimens enforced in the trials. Patients who received either the WATCHMAN™ device (Boston Scientific Corp., Natick, MA, USA) (n = 272) or AMPLATZER™ Amulet™ device (Abbott Laboratories, Chicago, IL, USA) (n = 197) were discharged with a variety of antithrombotic regimens such as single APT (35.8%), OAC alone (28.9%), DAPT alone (23%), or both OAC and APT (4.6%), whereas 7.7% were discharged with no drug therapy. The rate of major adverse events in this cohort was 20% at the mean follow-up time of 13 months; of these, 6.9% died, 4% had an ischemic stroke, 3.8% had a major hemorrhage, and 7.2% had thrombus detected on the device on follow-up LAA imaging.^28^ As only 72% of patients underwent follow-up LAA imaging, the device-related thrombus rate in this population is likely an underestimation.^28^ The clinical significance of device-related thrombi has been questioned.^29,30^ The higher ischemic stroke rate in patients with device thrombus in comparison with patients without (15.4% versus 3.2%)^28^ suggests prognostic significance. This reinforces the need for postimplantation LAA imaging, as the presence of a device thrombus could change the management protocols even in patients with high bleeding risks. Although the greatest potential benefit of LAAC would seem to be in patients unable to tolerate anticoagulation at all, LAAC devices used in this population may also have the greatest potential for harm. Until we directly compare LAAC versus placebo in this patient population in a randomized trial, we will not have accurate information for a good risk–benefit decision.

LAAC has not yet been compared in a head-to-head manner with novel OACs (NOACs), which have demonstrated an improved risk–benefit profile when compared with warfarin in terms of stroke risk; intracranial hemorrhage; and, in some cases, the risk of major bleeding.^13–16,31,32^ The comparison of LAAC and NOAC should also be based on randomized trials rather than on the extrapolation of data from PROTECT AF and PREVAIL. According to the manufacturer’s recommendations, patients receiving the WATCHMAN™ device (Boston Scientific Corp., Natick, MA, USA) will need long-term ASA therapy. Based on the Phase III Study of Apixaban in Patients with AF (AVERROES) trial, when used in patients thought to be unsuitable for OAC in conjunction with warfarin, apixaban was superior to ASA in preventing ischemic events, with similar bleeding rates.^33^ Based on this, perhaps patients who can tolerate long-term ASA might also tolerate long-term apixaban. Thus, use of the WATCHMAN™ device (Boston Scientific Corp., Natick, MA, USA) might not be the ideal answer.

In summary, LAAC devices are potentially promising additions to the armamentarium of treatment options for certain patients with AF. As it stands currently, LAAC devices offer an alternative to long-term anticoagulation with less protection against ischemic events but with the potential benefit of a decreased bleeding risk. An individualized risk–benefit analysis with regard to stroke risk, bleeding risk, the risks associated with an invasive procedure, and the long-term risk of a foreign object being placed in the left atrium should be weighed when determining the suitability of a particular patient to undergo the procedure. Finally, most data currently available are regarding a particular device; thus, it is possible that alternative designs such as the AMPLATZER™ Amulet™ device (Abbott Laboratories, Chicago, IL, USA) and LARIAT^®^ device (SentreHEART, Redwood City, CA, USA) as well as others currently in development may have significant advantages that will extend the reach and role of LAAC as a means to prevent stroke in this complex patient population.
